# Validation of Deep Learning-based Sleep State Classification

**DOI:** 10.17912/micropub.biology.000643

**Published:** 2022-10-04

**Authors:** Wei Chen, Xiaohui Zhang, Hanyang Miao, Michelle J. Tang, Mark Anastasio, Joseph Culver, Jin-Moo Lee, Eric C. Landsness

**Affiliations:** 1 Department of Neurology, Washington University School of Medicine, St. Louis, MO 63110, USA; 2 Department of Bioengineering, University of Illinois Urbana-Champaign, Urbana, IL 61801, USA; 3 Department of Radiology, Washington University School of Medicine, St. Louis, MO 63110, USA; 4 Department of Biomedical Engineering, Washington University School of Engineering, St. Louis, MO 63130, USA; 5 Department of Electrical and Systems Engineering, Washington University School of Engineering, St. Louis, MO 63130, USA; 6 Department of Physics, Washington University School of Arts and Sciences, St. Louis, MO 63130, USA; 7 Department of Neurology, Washington University School of Medicine, St. Louis, MO 63110, USA; 8 Department of Radiology, Washington University School of Medicine, St. Louis, MO 63110, USA

## Abstract

Deep learning methods have been developed to classify sleep states of mouse electroencephalogram (EEG) and electromyogram (EMG) recordings with accuracy reported as high as 97%. However, when applied to independent datasets, with a variety of experimental and recording conditions, sleep state classification accuracy often drops due to distributional shift. Mixture z-scoring, a pre-processing standardization of EEG/EMG signals, has been suggested to account for these variations. This study sought to validate mixture z-scoring in combination with a deep learning method on an independent dataset. The open-source software Accusleep, which implements mixture z-scoring in combination with deep learning via a convolutional neural network, was used to classify sleep states in 12, three-hour EEG/EMG recordings from mice sleeping in a head-fixed position. Mixture z-scoring with deep learning classified sleep states on two independent recordings with 85-92% accuracy and a Cohen’s κ of 0.66-0.71. These results validate mixture z-scoring in combination with deep learning to classify sleep states with the potential for widespread use.

**Figure 1. Evaluating the performance of mixture z-scoring followed by deep learning sleep classification:**
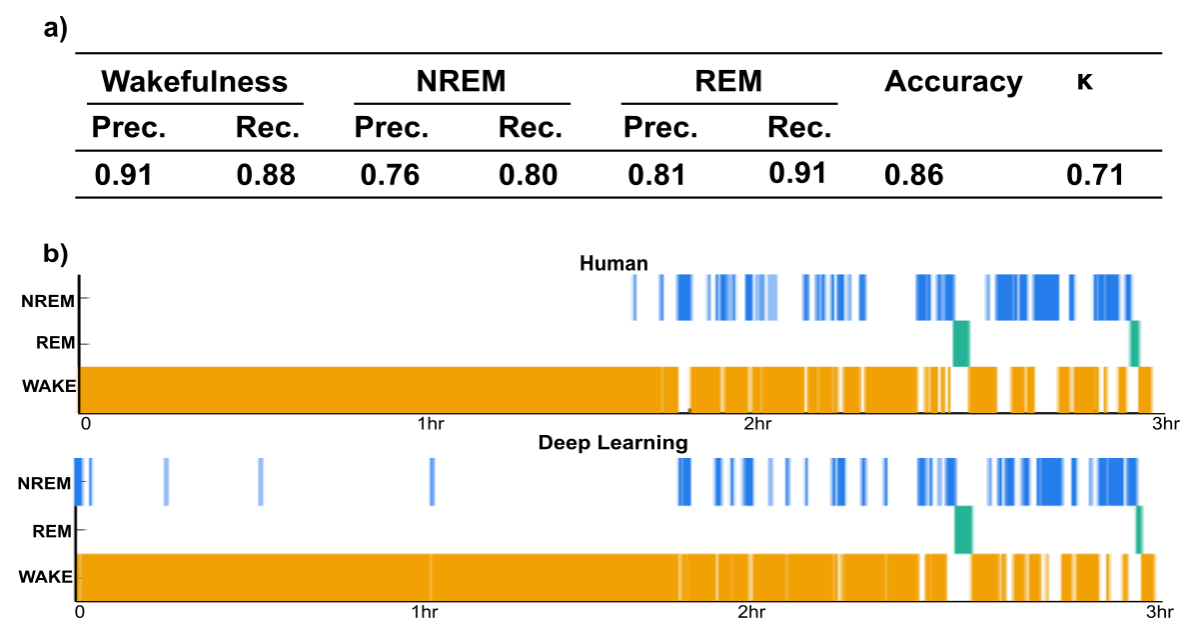
a) Metrics evaluating the performance of the algorithm on three sleep states and overall accuracy on the 20% test set. Prec., precision; Rec., recall. precision=TP/(TP+FP), recall=TP/(TP+FN), and accuracy=(TP+TN)/(TP+TN+FP+FN), where TP, TN, FP, FN are the numbers representing true positive, true negative, false positive, and false negative, respectively. b) Hypnogram of human scoring compared to deep learning classification of a single independent held-out recording.

## Description

Classification of rodent sleep recordings requires expert human researchers to manually score the EEG/EMG into various sleep states, at the cost of high labor and time bottlenecks. To overcome these limitations, machine learning methods were developed to automatically classify sleep states with accuracies ranging from 70% to 95% (Charbonnier et al. 2011; Libourel et al. 2015). Recently, deep learning methods to classify sleep states have reported accuracies up to 97% (Miladinovic et al. 2018; Barger et al. 2019; Yamabe et.al. 2019). While these deep learning methods have proven to be highly accurate when trained and validated on their own datasets, generalization to independent datasets has been difficult (Katsageorgiou et al. 2015). Generalizability across datasets is challenging because independent datasets are often collected under experimental (e.g., sleep deprivation, pharmacological manipulation, or disease states) or recording (e.g., variability between different genetic strains of mice, electrode placement, or the presence of artifacts) conditions different from the original training dataset. Thus, a pre-processing method that standardizes EEG/EMG recordings is needed prior to classifying sleep states by use of deep learning methods.

One way to standardize EEG/EMG recordings is through mixture z-scoring (Barger et al. 2019). To prevent systematic bias and preserve the distributional shifts that occur across datasets, mixture z-scoring calibrates each mouse’s recording using a small amount of labeled data. This standardization combined with a deep learning-based classification model has been successfully implemented and tested on a single dataset with an accuracy of 97% (Barger et al. 2019). Unfortunately, this method has not been validated on an independent dataset. The purpose of this study is to validate mixture z-scoring combined with deep learning of an EEG/EMG dataset collected under specific experimental (head-fixation) and recording (non-wild type mice with cranial plexiglass windows and EEG electrodes placed laterally on the skull) conditions. We find that despite experimental and recording conditions unique to this independent dataset, mixture z-scoring followed by deep learning classifies sleep state with a high level of accuracy. Therefore, this method holds promise for widespread use.

**RESULTS**

To validate mixture z-scoring combined with deep learning-based sleep state classification on an independent dataset, data from 11 mice undergoing sleep recordings and optical imaging in the head fixed position were trained and validated using the open-source software Accusleep. When applied to test data, mixture z-scoring combined with deep learning yielded an overall accuracy of 86% and a Cohen’s κ of 0.71 indicating substantial agreement (Figure 1a) (Landis and Koch 1977). For the individual sleep states the precision (recall) was 0.91 (0.88) for wakefulness, 0.76 (0.80) for NREM, and 0.81 (0.91) for REM (Figure 1a).

To further illustrate the ability of mixture z-scoring combined with a deep learning-based model to classify sleep states, two unseen 3-hour recordings were classified. As depicted by the hypnogram (Figure 1b), there was a substantial agreement in the temporal pattern (sleep cycles) of transitions between wakefulness, NREM, and REM between the human raters and the deep learning method with an accuracy of 92% and a Cohen’s κ of 0.71. For individual sleep states the precision (recall) was 0.94 (0.96) for wakefulness, 0.76 (0.67) for NREM, and 0.95 (0.87) for REM. For the other individual recording, the deep learning method yielded 85% accuracy and a Cohen’s κ of 0.66. Precision (recall) was 0.94 (0.86) for wakefulness, 0.65 (0.86) for NREM, and 0.96 (0.70) for REM. Together, these results show that sleep states classified by mixture z-scoring combined with deep learning-based model can achieve a high degree of accuracy (85-92%).

**DISCUSSION**

The purpose of this study was to assess the generalizability of mixture z-scoring combined with a deep learning model to classify mouse sleep states. When applied to an independent EEG/EMG sleep dataset, this approach successfully classified sleep states with high accuracy.

Accurate sleep state classification by deep learning often relies on large sample sizes and high-quality recordings. Previous attempts of applying machine learning algorithms to small-scale datasets (N=14 for a training set) yielded an accuracy of 91-96%, which is lower than the 94-97% accuracy achieved when applied to large-scale datasets (N=4,200) (Yamabe et al. 2019). In this study, mixture z-scoring combined with a deep learning-based model applied to a small-scale (N=11) dataset achieved an accuracy of 85-92%, but was not as high as the 97% previously reported when applied to a different dataset (Barger et al. 2019). One potential reason for the decreased accuracy may be due to the decreased size of the dataset compared to the original dataset (Barger et al. 2019). Another potential reason could be the lateral placement of the EEG electrodes used in this study. The EEG electrodes were placed laterally, compared to the typical somatosensory or motor cortex locations, to avoid obstruction of the field of view of the plexiglass window used for simultaneous optical fluorescence imaging. As a result, the electrodes were located adjacently to muscles attached to the skull and could have led to poorer signal quality, changes in the EEG spectral profile, and subsequent poorer performance of the model. Another cause of the disrupted signal could be due to the head fixed position of the mice during recordings (typically mice are freely moving during sleep studies). This unnatural sleeping position (mice prefer to sleep in a curled-up position) may have prevented a clear differentiation of the relatively decreased muscle tone of NREM and REM sleep in the EMG, potentially leading to decreased performance of the model. A fourth possible source of signal quality disruption is the inclusion of artifacts into the training of the sleep classification model. Often, artifacts will be removed prior to sleep-state classification model training. In this study, artifacts were not excluded when training the model in order to replicate real-world settings where artifacts are typically part of the recordings, which may have decreased the accuracy. To address potential causes for decreased accuracy, a future study using EEG electrodes placed in typical skull locations in a higher number of freely moving mice with all artifacts excluded is needed.

Manual scoring of EEG/EMG sleep recordings is laborious, requires highly-trained experts and is prone to inter-individual variability. Expert human scorers of EEG/EMG often only agree upwards of 92%, and the level of disagreement increases during sleep-wakefulness transitions (Loredo et al. 1999; Rosenberg et al. 2013). In our study, the largest source of disagreement between human scored EEG/EMG and the mixture z-scoring and deep learning model was also at transitions between wakefulness and NREM which is known to often contain a mixture of sleep states and historically has the lowest inter-rater reliability among human scorers (Merica and Fortune 2004). To avoid sleep-wake transition misclassifications, other studies have assigned sleep states based on a consensus of multiple experts independently scoring the same recording (Yamabe et al. 2019). Our study, however, used EEG/EMG recordings scored by a single sleep scoring expert (E.L.), increasing the possibility for human error with the subsequent decreased performance of the model. Future studies assessing the generalizability and accuracy of sleep state classification models may be improved by using datasets scored by a consensus of sleep-scoring experts.

Mixture z-scoring is designed to allow for generalizability across datasets by calibrating the deep learning model using representative samples of each state in the recording of each subject. However, the selection of representative samples could have had a significant influence on performance by biasing the deep learning classifier to look for similar samples within the recording. For example, NREM sleep is defined by the presence of slow waves and spindles whereas REM sleep can be phasic or tonic in humans and intermediate sleep with high amplitude theta activity can precede the onset of REM in rodents (Glin et al. 1991). In this study, 100 representative seconds per state (Wake, NREM, REM) were used for calibration. If some of these NREM/REM characteristics were over or underrepresented in the calibration, it may have biased classification toward or away from certain EEG/EMG characteristics. Alternatives in the future would be to increase the amount of representative scoring samples or to examine the effect of systematically varying which representative samples are used for mixture z-scoring.

In summary, applying mixture z-scoring combined with deep learning-based classification model using an independent dataset with unique recording and experimental conditions was able to classify sleep states with an accuracy of 85% to 92%. While this accuracy is not as high as previously reported methods, the accuracy of the model may have been limited by, the size of the dataset, changes in signal quality, human error, or the choice of samples used for mixture z-scoring calibration, which should be accounted for in future studies. Despite its limitations, this study will be useful to sleep researchers planning to implement machine learning models on their own experimental datasets using automated methods that can achieve ~90% accuracy. Future studies replicating these findings across multiple independent datasets would further strengthen the claim that mixture z-scoring followed by deep learning is a valid methodology and would encourage widespread use.

## Methods

This study was approved by the Washington University School of Medicine Institutional Animal Care and Use Committee and performed in accordance with National Institutes of Health Guide for the Care and Use of Laboratory Animals. A total of 12 transgenic mice (12-16 weeks of age) expressing GCaMP6f in excitatory neurons (driven by a Thy1 promotor) acquired from Jackson Laboratories (JAX Strain: C57BL/6J-Tg(Thy1-GCaMP6f)GP5.5Dkim; stock: 024276) were used. Mice were housed in 12:12 hour light/dark cycles with lights on at 6:00 AM and given ad libitum access to food and water.

**Experimental Design**

EEG/EMG data of head-fixed mice was collected during wakefulness, NREM (Non-rapid Eye Movement) sleep, and REM (Rapid Eye Movement) Sleep reported in a previously published dataset (Landsness et al. 2021). Prior to data collection, mice were acclimated to head fixation while secured in a black felt hammock for one to three sessions ranging from 30 to 180 minutes until the EEG/EMG signals showed the presence of sleep. Once sleep was established in the head-fixed position, the mouse then underwent a three-hour undisturbed recording session. All recordings occurred between 9:00 AM and 1:00 PM during the mice’s normal sleeping hours to maximize the chance of recording sleep. After 12 three-hour recordings were collected, human experts scored recordings in 10-second epochs as wakefulness, NREM, or REM as previously described (Zhang et al. 2021).

**Surgical Techniques**

As part of the previously published dataset, mice had been fitted with EEG/EMG electrodes and plexiglass windows to allow simultaneous EEG/EMG and optical fluorescence imaging (Landsness et al. 2021). Following scalp retraction, EEG pins (Newark Electronics, catalog # 89H8939) were placed at the surface (0.7 mm cranial burr holes) of the brain overlying the somatosensory cortex (-7mm posterior to bregma, and +4mm lateral to bregma) and fixed with Fusio dental cement. An EEG pin placed on the surface of the cerebellum served as a bipolar reference. To record muscle activity, two 23-gauge stainless steel needles were inserted bilaterally into the neck muscles. A plexiglass head cap was fixed with a translucent adhesive cement (C&B-Metabond, Parkell Inc., Edgewood, New York) to allow for chronic, repeated imaging (Wright et al. 2017). Following surgery, mice were allowed to recover for 7 days in their home cages.

**Deep learning and mixture z-scoring**

Eleven, 3-hour EEG/EMG recordings were manually scored in 10-second epochs for a total of 11,880 epochs. Then the pooled 11,880 epochs were randomly split into a training (80%), validation (10%), and test (10%) set. The EEG/EMG spectrogram of the pooled data of the training set was used to train the convolutional neural network (CNN) deep learning model described in Barger et al. From the training set, 100 seconds of expert-scored wakefulness, NREM, and REM from each record underwent mixture z-scoring to create calibration files specific to each subject. Then the remaining test set from each record was classified by the CNN. To further evaluate the performance of deep learning sleep state classification on a recording that was previously unseen by the model, two complete 3-hour recordings from two independent mice were withheld from the initial training. These recordings had mixture z-scoring applied to 100 seconds of each sleep state and were then classified with the CNN.

Deep learning sleep classification and mixture z-scoring was implemented using the open-source software program AccuSleep (custom MATLAB package). https://github.com/zekebarger/AccuSleep

**Statistical comparisons**

Metrics including precision=TP/(TP+FP), recall=TP/(TP+FN), and accuracy=(TP+TN)/(TP+TN+FP+FN), where TP, TN, FP, FN are the numbers representing true positive, true negative, false positive, and false negative, respectively, were used to evaluate the model performance. The Cohen’s kappa statistic, κ, was computed to assess the inter-rater reliability between manual EEG/EMG-based scoring and mixture-z scoring with deep learning-based classification results. The kappa statistic is thought to be a more robust measure than percent agreement, and a kappa magnitude between 0.61 and 0.80 indicates a substantial agreement between the two raters (Landis and Koch 1977; Zhou et al. 2021).
